# Recent Updates on Mouse Models for Human Immunodeficiency, Influenza, and Dengue Viral Infections

**DOI:** 10.3390/v11030252

**Published:** 2019-03-13

**Authors:** Vinodhini Krishnakumar, Siva Sundara Kumar Durairajan, Kalichamy Alagarasu, Min Li, Aditya Prasad Dash

**Affiliations:** 1 Department of Microbiology, School of Life Sciences, Central University of Tamilnadu, Tiruvarur 610 005, India; kvinodhini@cutn.ac.in; 2 Dengue/Chikungunya Group, ICMR-National Institute of Virology, Pune 411001, India; alagarasu@gmail.com; 3 Neuroscience Research Laboratory, Mr. & Mrs. Ko Chi-Ming Centre for Parkinson’s Disease Research, School of Chinese Medicine, Hong Kong Baptist University, Kowloon Tong, Hong Kong, HKSAR, China; 4 Central University of Tamilnadu, Tiruvarur 610 005, India; apdash@gmail.com

**Keywords:** infectious diseases, human viruses, mouse models, knockout mice, transgenic mice, humanized mice, HIV, influenza, dengue

## Abstract

Well-developed mouse models are important for understanding the pathogenesis and progression of immunological response to viral infections in humans. Moreover, to test vaccines, anti-viral drugs and therapeutic agents, mouse models are fundamental for preclinical investigations. Human viruses, however, seldom infect mice due to differences in the cellular receptors used by the viruses for entry, as well as in the innate immune responses in mice and humans. In other words, a species barrier exists when using mouse models for investigating human viral infections. Developing transgenic (Tg) mice models expressing the human genes coding for viral entry receptors and knock-out (KO) mice models devoid of components involved in the innate immune response have, to some extent, overcome this barrier. Humanized mouse models are a third approach, developed by engrafting functional human cells and tissues into immunodeficient mice. They are becoming indispensable for analyzing human viral diseases since they nearly recapitulate the human disease. These mouse models also serve to test the efficacy of vaccines and antiviral agents. This review provides an update on the Tg, KO, and humanized mouse models that are used in studies investigating the pathogenesis of three important human-specific viruses, namely human immunodeficiency (HIV) virus 1, influenza, and dengue.

## 1. Introduction

Infectious diseases caused by human pathogenic viruses remain a huge threat to global health. An estimated 37 million persons are living with human immunodeficiency virus (HIV) infections [1]. More than 90 million new cases of influenza occur every year in children aged less than five years; of these, 20 million are acute lower respiratory infections and one million are severe acute lower respiratory tract infection (ALRI) cases [2]. The mechanisms of how these pathogenic viruses cause human disease differ widely. Using cell culture systems, the effect of these pathogenic viruses on the biology of the cell can be investigated. Organotypic cultures provide an additional opportunity for studying viral spread and tissue remodelling, but offer limited potential for studying host-pathogen interactions [3]. Virologists attempt to understand how the replication of a virus within a host induces an infection process which may be associated with symptoms, disease, or a sub-clinical infection. Animal models are important for investigating the exact *in vivo* circumstances, and furthermore, are fundamental for developing counter measures against infectious diseases.

Mouse models are the preferred animal models for studying human diseases due to their ready availability, low cost, and convenient husbandry requirements. In addition, species-specific reagents can be used and certain species are amenable to genetic manipulation. With mouse models, the host response to infection can be analyzed in detail and the effectiveness of drugs and vaccines, as well as their safety, can be assessed. However, there are certain drawbacks in the utilization of mouse models for research and in the translation of this animal data to humans. Viruses exhibit high species-specificity and specific tropism, depending on the presence or absence of viral entry receptors, as well as specific innate immune response factors in the mouse cells. Hence, most mouse models show reduced susceptibility to infection by human viruses and do not show disease symptoms. Indeed, with any human virus, investigating immune responses *in vivo* is arduous, even when utilizing appropriate animal models.

Genetically engineered mouse (GEM)/Transgenic mouse (Tg) models enable researchers to perceive the mechanisms behind viral pathogenesis. Advances in transgenic technology have enabled the analysis of the role of the specific viral proteins found in affected cells and the mechanisms of host susceptibility and disease symptoms. Researchers have developed efficient tools to investigate immune responses to viral infection *in vivo*. Mice with germline genetic modifications have revealed the mechanisms involved in viral pathogenesis and anti- viral immune responses. Thus, for instance, to analyze virus-specific T-cell responses, Tg mouse models that express T-cell receptor (TCR) transgenes specific to viral proteins have been widely employed. These transgenic mice include mice expressing a transgenic TCR specific to influenza virus hemagglutinin (HA) in the context of the major histocompatibility complex (MHC) class II molecules. These mice, when infected with mouse adapted strains of influenza, show both a CD4+ and CD8+ T cell response, and thus have been used to study the role of CD4+ T cells in the activation of CD8+T cells [4,5]. Furthermore, many human viruses like hepatitis viruses, polioviruses, papillomaviruses, HIV, and measles are not infectious to wild-type mice, but the transgenic mice expressing specific human receptors that aid in the entry of those viruses are susceptible to many of these viruses. These susceptible Tg mice can be used to evaluate the virus’ pathogenesis as *in vivo* models [6]. Although the transgenic mice are useful in evaluating the involvement of key viral proteins in disease pathogenesis, they cannot recapitulate the entire disease symptom complex as in the host.

Knockout (KO) mice devoid of specific genes have been used to identify and evaluate the cellular and molecular entities involved in the adaptive and innate immune responses that are important in controlling viral infections. These KO mice include: TCR-B KO mice lacking alpha beta T-cells [7]; recombination activation gene (RAG)-1 and RAG-2 KO mice that are devoid of mature B and T cells [8]; KO mice for type I and type II interferons (IFNs) or IFN receptors which lack an antiviral response [9]; KO mice for immune receptors, such as Toll like receptors (TLRs), retinoic acid-inducible gene-I (RIG-I)-like receptors (RLRs) and nucleotide-binding oligomerization domain (NOD)-like receptors (NLRs) [10]; and KO mice for transcription factors, such as Signal Transducer and Activator of Transcription (STAT) molecules [11] and IFN regulatory factors (IRFs) [12] that participate in linking signals from receptors to downstream kinases and transcription factors [10,11].

**Figure 1:**
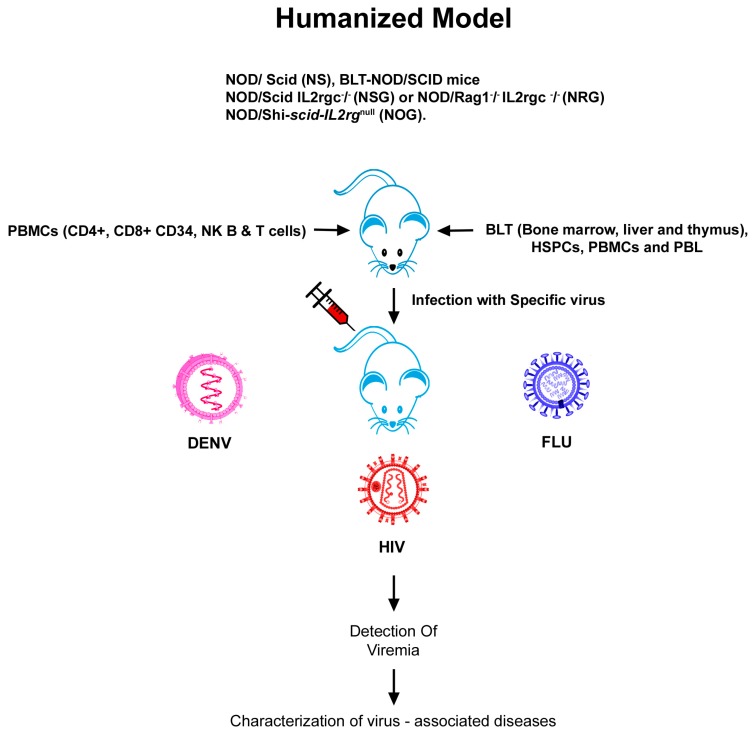
Humanized mouse model represents the engraftment of human cells and tissues into immunodeficient mice. Humanized mouse model representing infection with HIV, FLU, or DENV.

Humanized mouse models are an advanced approach for evaluating the human immune response to viral pathogens, as well as to unravelling the pathogenic mechanisms of viral diseases. Severely immunodeficient mouse strains are engrafted with human cells and/or tissue xenografts to produce humanized mice (Figure 1). The major breakthrough has been the discovery of mice with mutations in the gene coding for the protein kinase DNA-activated catalytic polypeptide mutation (Prkdc). These mice, called severe combined immunodeficient (SCID) mice, allow the poor engraftment of human fetal tissues, human peripheral blood mononuclear cells (PBMCs), and haematopoetic stem cells (HSCs). Further, mice with mutations in the RAG- 1 or 2 genes (Rag1null or Rag2null, respectively) also have T and B cell deficiency. Backcrossing of mice with the Prkdc^SCID^ mutation (CB17-Prkdc^SCID^) to mice on the NOD (non-obese diabetic) background resulted in NOD.CB17-Prkdc^SCID^/J mice, which allowed for the improved engraftment of HSCs, but they developed thymic lymphomas and had a short life span. The production of immunodeficient mice with mutations in the interleukin (IL) 2 receptor common gamma chain (IL2rγnull) allows for higher rates of the engraftment of HSCs due to the absence of natural killer (NK) cells [13]. When IL2rγnull mice were merged with Prkdcsid or SCID mice or with recombination of activating genes (Rag) 1 or 2 null mice (Rag1null or Rag2null), the resulting hybrids lacked adaptive immunity and expressed acute deficiencies of innate immunity, besides lacking murine natural killer (NK) cells and allowing for the engraftment of human cells at high levels [14,15,16]. Adopting this same approach, mice harbouring human hematopoietic cells, myoblasts, neurons, hepatocytes, and different kinds of epidermal derivatives have been raised. Invariably, these humanized mice are susceptible to a spectrum of viruses, and humanized mice can be used to investigate the diseases caused by these viruses (e.g., dengue and HIV-I in mice with a human immune system).

Three strains of immunodeficient IL2rγnull mice, namely NOD Cg-PrkdcidII2rgtm1WjI (NSG), NOD Cg- PrkdcsidII2rγtm1Sug (NOG), and C;129S4-Rag2tm1FvII2rγtm1F1v (popularly denoted as BALB/c-Rag2null, IL2rγnull, or BRGS mice with the NOD SIRPa variant) [14,16,17,18,19], are commonly used today. NOG mice possess a truncated cytoplasmic domain of the gamma chain, but lack the signalling domain, while NSG and BRG mice completely lack the gamma chain. The biological responses of humanized IL2rγnull mice, when engrafted with human tissues, cells, and immune structures, recapitulate those observed in humans better than any previously known models of humanized mice [14,16,17,18,19].

Three common approaches are used to engraft human immune systems into immunodeficient IL2rγnull mice. The first model, denoted as the human peripheral blood leuckocytes (Hu-PBL)-SCID model, is generated by the injection of human PBMCs followed by the engraftment of human CD3+T-cells after the first week. These mice develop a lethal xenogeneic graft-versus-host disease (GVHD) and their life span is short [16,17,18,19]. The second model, referred to as the humanized SCID repopulating cell (Hu-SRC)-SCID model, is created by the intravenous (IV) or intrafemoral injection of precise SRC, i.e., human CD34+ HSCs acquired from bone marrow, fetal liver, cord blood, or peripheral blood. This model (referred to as HSC engrafted) affirms engraftment of the entire human immune system. The third model is the bone marrow/liver/thymus (BLT) model, developed by transplantation of the human fetal liver and thymus below the kidney and IV injection of autologus fetal liver HSCs [20,21]. Similar to the Hu-SRC-SCID model, all lineages of human hematopoietic cells grow. In spite of the development of a healthy mucosal human immune system by BLT mice, the human T cells are educated in an autologous human thymus and are human leuckocyte antigen (HLA) restricted. One of the salient caveats of the BLT model is that, in most laboratories, the mice develop a dissipating GVHD-like syndrome that narrows the window for experimentation [16,17,18,19].

**Figure 2:**
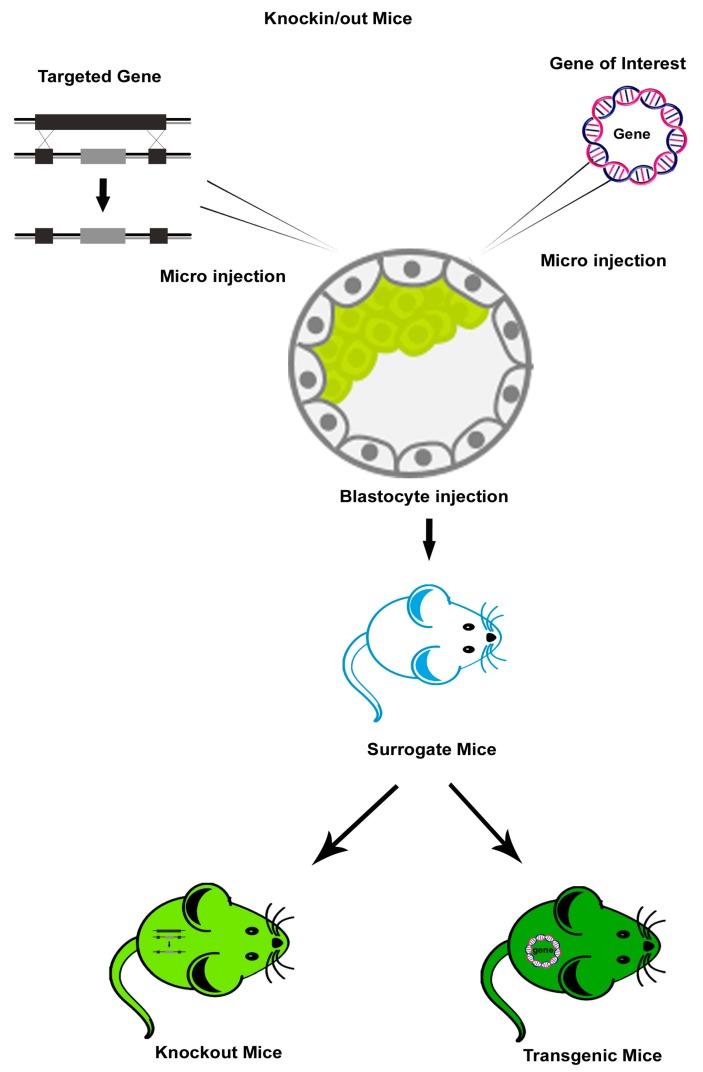
Generation of transgenic (Tg) mice and knock out (KO) mice. The embryonic stem cells (ES) with inserted or disturbed specific gene of interest are microinjected into the mouse blastocyst, which allows the development of a new Tg or KO progeny.

Every model is endowed with specific advantages and restrictions; hence, selecting an appropriate model for a viral disease of interest is of paramount importance. Schematic representation of the generation of transgenic, KO, and humanized mouse models using genetice engineering is depicted in Figure 1 and Figure 2. The most widely used and recently reported KO, Tg, and humanized mouse models of HIV, influenza, and dengue are discussed and presented in Table 1, Table 2 and Table 3.

## 2. Human Immuno Deficiency Virus

Acquired immunodeficiency syndrome (AIDS) is a spectrum of conditions caused by infections of HIV type 1 (HIV-1) and HIV type 2 (HIV-2). HIV-1 is a major contributor to the global AIDS pandemic. More than 70 million individuals are living with HIV and 940,000 affected individuals died due to AIDS in 2017 [1]. The introduction of antiretroviral therapy (ART) is one of the most significant inventions in modern medicine, providing effective virus suppression and radically enhancing the quality of life and life span of HIV patients; however, the treatment needs to be continued for life. The major barriers posing a stumbling block to developing an effective vaccine and treatment methods are: (i) HIV’s inherent ability to mutate frequently, specifically its external envelope glycoproteins, resulting in the escape of immune variants; (ii) persistence of HIV in resting cells, which leads to incomplete suppression; and (iii) HIV infection in humans induces immune suppression, resulting from the massive and continuous depletion of CD4+ T-cells. Notwithstanding these barriers, encouraging developments in an HIV vaccine have been made, and various promising numbers of vaccines are being developed. So far, animal models are the best approach we have to deciphering the mechanisms of pathogenesis, disease progression, latency, and emergence of drug resistance mutations. Moreover, animal models are important for testing the efficacy of vaccines and anti retroviral drugs [22].

### 2.1. Knockout Mouse Models of HIV

Recently, retroviral restriction factors have drawn the attention of researchers. HIV-1 infection and replication is restricted by a number of host proteins, known as restriction factors, that interfere with the critical processes involved in the life cycle of retroviruses. Four of these are sterile alpha motif domain-containing protein 1 (SAMHDI) [23,24,25], apolipoprotein B mRNA editing enzyme catalytic subunit 3G (APOBEC3G) [26], viperin [27], and tetherin [28]. These restriction factors inhibit viral replication and the production of new progeny virions by preventing reverse transcription, by inducing viral DNA to mutate, and by promoting TLR 7- and TLR9-mediated synthesis of type I IFN by plasmacytoid dendritic cells (pDCs).

Mostly, these restriction factors are conserved and orthologous in mice. KO mouse models (Table 1) help researchers to validate *in vitro* results obtained from cultured cells and to gain a better understanding of the biology of restriction factors. *In vivo* studies of mouse models provide greater insight into the mechanisms by which the proteins act to restrict viral infection and replication. Wittmann et al. [23] and Bloch et al. [29] showed that SAMHD1 in the KO mouse model blocks retroviral infection and that endogenous mouse SAMHD1 restricts not only HIV-1, but also MLV reporter virus infection at the level of reverse transcription in primary myeloid cells. Intracellular dNTP levels and SAMHD1 phosphorylation in different T-cell types are the two most important factors affecting the antiviral activity of murine SAMHD1. They also reported that the antiviral restriction in a mouse by SAMHD1 is mechanically similar to that in humans in terms of dNTP hydrolase activity and cell-dependent phosphorylation. This study provided evidence that the SAMHD1 KO mouse is a valuable tool for studying the replication of different viruses, including retroviruses, retro elements, and DNA viruses [23,29].

### 2.2. Transgenic Mouse Models of HIV

To overcome the barrier to HIV entry and infection in mouse cells, Browning et al. [30] generated human CD4 and CCR5 double Tg mice (hu CD4/CCR5 Tg mice). Though human CD4 and a chemokine receptor CCR5 helped HIV-1 to enter mouse cells, establishment of further *in vivo* infection and replication in these human CD4/CCR5 Tg mice was not observed due to other cellular blocks [31]. Tg rats with human CD4 and CCR5 also supported HIV infection; however, as in double Tg mice, spread of the virus was blocked [32]. Transient trans complementation of human Tat interacting protein Cyclin T1 elevated HIV gene expression, suggesting novel ways of enhancing HIV infection and replication in rat models [32]. Multiple research groups have constructed different Tg mice that express the whole or partial HIV-1 genome. They include Tg mice expressing the HIV-1 genome with modified long terminal repeats (LTR), resulting in cataracts, weeping eyes, and wasting in Tg mice [33]; 3′ half of the HIV genome, causing severe nephropathy [34]; and expression of HIV-1 Tat or Nef proteins, leading to epidermal hyperplasia [35]. Hanna et al [36] constructed Tg mice by expressing entire HIV coding sequences in T-cells and cells of the macrophage/dendritic cell lineage under the control of a human CD4 promoter flanked by an enhancer of mouse CD4. Tg mice expressing defective provirus with gag and pol genes deleted or expressing individual HIV-1 genes have been shown to develop various pathologies resembling those in humans [37]. *In vivo*, a negative factor (Nef) was observed to promote viral replication and pathogenicity. Tg mice expressing entire coding sequences of HIV-1 (CD4/HIV^WT^) or HIV-1 Nef alone (CD4C/HIV^Nef^) under the control of a CD4 promoter in HIV-1 target cells showed human AIDS-like symptoms [38]. Inducible HIV-1 type Nef Tg mice (Table 2) were generated using a tetracycline-inducible system to prevent the developmental defects due to the continuous expression of Nef in CD4C/HIV^nef^ Tg mice. The Nef gene was induced in (CD4C/rtTA X TRE/HIV^Nef^) or (CD4C/rtTA2^S^-M2 X TRE/HIV^Nef^) double-Tg mice upon doxycycline (DOX) treatment, and these mice developed disease similar to that seen in constitutively Nef-expressing 

CD4C/HIV^Nef^ Tg mice. In the absence of lymphopenia, CD4^+^ T-cell activation was observed only in Nef-expressing T-cells, but not in T-cells that do not express Nef. This model helped in understanding the functions of Nef [39]. A Tg mouse that expresses gp120 of HIV-1 in the astrocytes developed neuropathological features similar to those observed in AIDS patients with neurological symptoms and can be utilized in neuro AIDS research [40]. The same mouse model expressing gp120 of HIV-1 has been utilized to demonstrate the pathological role of CCR5 in HIV-associated brain injury. Genetic ablation of CCR5 inhibits microgilial activation (hallmark of brain pathology) in a Tg mouse model expressing envelope glycoprotein 120 (gp120) that uses CXCR4 as a receptor to induce HIV-associated brain injury. A pharmacological blockade of CCR5 and the acute phase protein lipocalin prevent neurotoxicity in CCR5-deficient mice expressing gp120 of HIV-1 [41]. Different research groups have used Tg mice extensively for studies related to HIV-associated nephropathy. To list a few, Tg26 and its variants, as well as CD4/HIV Tg mice and variants, have been used [42]. Tg26 is a transgenic mouse which expresses seven of the nine HIV proteins under the control of a viral LTR promoter. Since the DNA expressed is replication deficient, it randomly integrates into the host genome, and transcription of these genes takes place. Tg26 is used to study the long-term effect of HIV proteins on the host. Tg mouse models which express the entire genome or selected genes of HIV have given productive results and information on pathogenesis [42].

### 2.3. Humanized Mouse Models of HIV

Humanized mice have provided the opportunity to study HIV entry and infection, virology, latency, and disease progression. They are good and convenient models for testing prophylactic drugs and anti-HIV antibodies. HIV infection, replication, and pathogenesis can be studied *in vivo* using Hu-PBL-SCID mice. Human disease pathogenesis can be studied using HSCs-engrafted and BLT models. BLT models can be infected with either CCR5 or CXCR4-tropic HIV strains to study HIV mutations and how they escape from the CD8^+^ T-cell response [43,44,45,46]. NOD-SCID BLT humanized mice were used to study cell-to-cell transmission and retroviral spread *in vivo*; their results provided new insights and novel approaches for blocking the spread of viruses [47]. NOD-SCID BLT models have been helpful in understanding the utility of ART in decreasing the transmission of HIV from infected individuals to their partners. Studies of HIV-infected NSG-BLT mice revealed that ART significantly suppresses HIV in cervico-vaginal secretions and restores the CD4^+^ and CD8^+^ T-cell numbers [48]. A rapid rebound of viremia was also demonstrated when ART was discontinued in NSG-BLT mice [48]. Despite the many other uses of BLT-humanized mice, the development of GVHD in these mice limited their use for HIV cure studies until the development of C57BL/6 *Rag2^−^/^−^γc ^−/−^CD47^−/−^* triple knockout (TKO)-BLT mice. Lavender et al. [49] reported that, in TKO-BLT mice, HIV-1 latency can be maintained over unlimited periods when the mice are on ART, and quick viral recovery occurs following therapy removal. Compared to other BLT models, TKO-BLT mice offer a sufficient time to investigate the effects of extended periods of ART (15–18 weeks) on the latent reservoir and it delays in recrudescence in HIV-1 cure studies [49,50]. 

Advancement in the technology and understanding of the usage of animal models has enabled researchers to explore the establishment of stable, integrated, and non-productive states of HIV infection in individual cells, termed HIV latency; this latency has been the primary barrier to the development of an HIV cure, and is the most studied aspect of the virus. Brooks et al. [51] used SCID-hu thy/lv mice to demonstrate HIV latency by injecting HIV into human thymic organoids that develop in these animals. Following the above model and strategy, different studies have reported reconstituting many types of tissues in different humanized mice (human thymus and liver mice (Thy/Liv mice); human CD34+ HSCs (huCD34 mice), or both BLT mice). NOD/ SCID (NS), NOD/SCID IL2rgc^−/−^ (NSG), or NOD/Rag1^−/−^ IL2rgc ^−/−^ (NRG)). A humanized mice model reconstituted with different tissues and organs appears to be an ideal model to study the effect of ART on systemic HIV infection [52,53,54,55,56]. Upon ART treatment, huCD34 and BLT humanized mice (Table 3) tissues reconstituted with human hematopoietic lineages resulted in the repression of viral replication, reduction in plasma viral RNA (vRNA) below the detection limit, and maintainence or preservation of the CD4+ T-cell counts [45,46,47,48,49,50,51,52,53,54,55,56,57]. However, ART could not absolutely remove HIV infection, and the presence of latent viruses has been demonstrated in huCD34-NSG humanized mouse models [46,57]. The newer generation of T-cell-only mice (TOM) or myeloid-only mice (MOM), developed by reconstituting specific hematopoietic cell lineages, allows investigators to study the individual contribution of T-cells or macrophages to HIV persistence *in vivo*. NSG-hu thy/liv mice were generated by implanting human fetal thymus and liver tissues under the kidney capsule. They are scientifically reconstituted with T-cells alone and not with any other lymphoid lineages like monocytes/macrophages, B cells, or DCs. These mice were susceptible to HIV-1 infection and did not develop any signs of GVHD. Following combination ART, HIV latent reservoirs and resting CD4+ T-cells were observed [58,59]. This clearly indicates that TOM humanized models can be used to study HIV latency. In addition to studies with these models, there are reports suggesting that macrophages can serve as HIV-1 reservoirs [58,60]. ART treatment of HIV-infected MOM mice suggested that the half-life of the infected macrophages from MOM mice is shorter than that of infected T-cells in BLT mice [60]. The seemingly unlimited ability to transplant human immune cells into immunodeficient mice has led to the development of a wide array of techniques and assays. One prominent example is the *in vivo* viral outgrowth assay that quantifies the latent HIV reservoir. This assay not only validates the results of *in vitro* latent viral detection, but also quantifies the viral content with a greater sensitivity [61,62]. Improvements with humanized mice are endless. Satheesan et al. [63] used a human HSC-engrafted NSG humanized model (hu-NSG) to investigate and demonstrate HIV latency *in vivo*. They demonstrated the ability of HIV-infected human cells from HIV-infected hu–NSG mice on combinatorial antiretroviral therapy (cART) to act as a latent HIV reservoir. This model is an attractive alternative to humanized BLT mice and SCID-hu-thy/liv mice, which require the surgical manipulation and reconstitution of immune cells and tissues models to study HIV latency and latent T-cell reservoirs [63]. Thus, mice models have immensely contributed to the improved understanding of HIV pathogenesis, persistence, prophylactic and therapeutic intervention; they could be a vital tool in the eradication of HIV.

## 3. Influenza Virus

Influenza A pandemics have caused considerable disease and deaths. While influenza A and B cause only acute febrile illness in a majority of infected people, people at high risk, including pregnant women, children below the age of five years, elderly people aged more than 65 years, and persons with chronic medical conditions, can develop severe disease which may be lethal [64]. Influenza infections may promote and intensify numerous conditions like chronic obstructive pulmonary disease (COPD) [65] and, asthma [66]; They can increase the risk of a cerebro-vascular accident and myocardial infarction [67], and fetal loss in pregnant women [68]. Though antiviral therapies, drugs, and vaccines are available, influenza viruses still threaten humans and animals all over the world. The diversity of the virus enables it to have multiple hosts, while antigenic drift and shift play major roles in the wide occurrence of epidemics and pandemics [69,70,71]. Mouse models have played a significant role in delineating the pathogenesis of influenza and in the development of vaccines and therapeutics against the different types of the virus. Laboratory inbred strains of mice, such as BALB/c, C57BL/6, and DBA/2j, can be infected with mouse adapted strains of influenza. DBA/2j is highly susceptible to non-adapted influenza strains [72,73,74]. The most common symptoms exhibited by the infected mice include hypothermia, anorexia, and weight loss. However, depending on the dose and the strains of the influenza virus used, the infection might be lethal. The most commonly used mouse adapted strains of influenza include H1N1 influenza A/Puerto Rico/8/34 (PR8) or H1N1 influenza A/WSN/1933 (WSN). However, certain strains, such as the 1918 H1N1 pandemic influenza A and the 2009 H1N1 pandemic influenza A, do not require adaptation to infect mice [72,73]. The lack of suitable small-animal models for studies of influenza pathogenesis and for the development of vaccines and antivirals is one of the most serious obstacles to progress in research.

### 3.1. Knockout Mouse Models of Influenza A

KO mice models have been widely used in understanding the two most important aspects of influenza virus, namely: (i) how the early inflammatory response to influenza virus leads to the progression of lung diseases; and (ii) how reassortment and parental strains differ in virulence. IL-1R1^−/−^ mice infected with influenza virus were used to demonstrate the role of IL-1α and β in the pathogenesis of influenza. The study revealed that IL-1 contributes to the recruitment of CD4+ T cells to the site of infection and enhances IgM production, but also causes acute pulmonary inflammation [75]. Studies on IL-18^−^/^−^ mice revealed the importance of IL-18 in restricting influenza virus replication in lungs by augmenting the NK cell response [75,76]. Since IL-1 and IL-18 are produced by macrophages through the caspase-1 pathway, Thomas and his group in 2009 investigated the role of cryopyrin, an NLR, in inducing caspase-1 using cryopyrin^−^/^−^ and caspase 1^−^/^−^ mice. Cryopyrin and caspase-deficient mice succumbed to infection, and their demise was attributed to the reduction in pro inflammatory cytokines which play a central role in innate immunity and in moderating lung pathology in influenza pneumonia [77]. Mice models knocked out for multiple genes can be used for studying the combined effects of different molecules on the influenza pathogenesis. TKO mice (Table 1) deficient in the three signalling receptors, namely tumor necrosis factor (TNF)-R1, TNF-R2, and IL-1-RI, were used to determine the combined contribution of the TNF and IL-1 inflammatory response to H5N1 influenza A infection. Triple mutant C57BL/6J x 129S background mice [IL1-RI single cytokine receptor knock-out mice (IL1-R KO), C57BL/ 6J x 129Sv -*Il1r1^tm1Roml^*/J), and single cytokine receptor TNFR1 KO mice (TNF-R KO, C57BL/6J x 129Sv-*Tnfrsf1a^tm1Imx^ Tnfrsf1b^tm1Imx^*/J)] infected with H5N1 influenza A were observed to have longer survival, less morbidity, reduced lung inflammation, and a diminished cytokine response compared to wild-type mice. These results strengthen the suggested hypothesis that TNF and IL-1 contribute to the pathogenesis of H5N1 influenza A virus [78]. The anti inflammatory role of fatty acid binding protein 5 (FABP5) in H1N1 influenza A virus infection has been demonstrated using FABP5^−/−^ mice. Infection of C57BL/6J background mice with H1N1 influenza A virus from which the FABP5 (FABP5^−/−^) gene had been deleted revealed that FABP5 deficiency augments excessive oxidative damage, lipid peroxidation, and inflammation [79].

The emergence of new pandemic viruses is mainly due to genetic mutations and reassortments. A reassortant seasonal H3N2 influenza A virus containing the PA gene of highly pathogenic avian influenza H5N1 influenza A caused severe pneumonia in *Casp 1*^−/−^ mice [80]. Knockout mice models have been utilized to discern differences in the pathogenicity between reassortment and parental strains of influenza virus differences that may not be easily observed in wild-type mice.

### 3.2. Transgenic Mouse Models of Influenza A

Highly pathogenic avian H5 and H7 influenza A viruses continue to cross the species barrier and infect humans, causing diseases. Hence, they pose a constant threat and give rise to devastating pandemics [81,82]. Tg mice are powerful tools to identify the antiviral power of different human proteins that act as effective species barriers and also to identify the changes required in these avian viruses to overcome the species barriers. The IFN-regulated MX1 gene plays a major role in providing an effective innate immune response to control the influenza virus [83]. Tg mice expressing the entire human MX locus have been used to demonstrate that those Tg mice (Table 2) exhibit a higher resistance to H5 and H7 influenza A viruses, but are susceptible to H1N1 and H3N2 influenza A viruses. Moreover, it was also shown that engineered avian H7N7 influenza virus with signature mutations found in human viruses was able to overcome the resistance conferred by the Tg mice [84]. 

With the help of Tg mice, researchers can implement new strategies against the influenza virus. Wang et al. [85] came up with a novel anti-influenza strategy involving short-hairpin RNA (ShRNA) to disrupt the activity of hemagglutinin (HA) of influenza A virus, thereby inhibiting the virus. They developed a Tg mouse expressing an shRNA that specifically targets the conserved sequence of the HA of influenza A virus. The Tg mice displayed a constant ability to reduce influenza A virus infection and replication, suggesting the utility of an shRNA-based approach to prevent and control influenza virus infections in animals [85]. 

Tg mice expressing the MHC class I allele, HLA-A*02:01, were used to study the CD8+ T cell response to a heat inactivated H7N3 influneza A vaccine and to a modified vaccinia Ankara vectored vaccine expressing influenza virus epitopes. The studies reported that CD8+ T cells specific to immunodominant and subdominant epitopes were elicited, indicating the potential of the vaccine candidates [86,87]. Tg mice expressing the MHC class II allele, HLA-DR3, were used to evaluate the effect of an epitope-based vaccine candidate expressing cross conserved H1N1 influneza A CD4+ T cell epitopes and it was found that the viral load was reduced when the vaccinated HLA-DR3 expressing mice were challenged with pandemic 2009 H1N1 influenza A [88]. Therefore, Tg mice expressing HLA class I and class II alleles can be utilized in evaluating the CD8+ and CD4+ T cell responses to different influenza A vaccine candidates.

### 3.3. Humanized Mouse Models of Influenza A

Humanized mice models permit the testing of novel therapeutic approaches to control influenza, the identification of critical epitopes for vaccine development, the preclinical analysis of the human immune response against influenza vaccines, and the evaluation of vaccine cytotoxicity. Direct manipulation or boosting of the host immune system is being considered as an alternative therapeutic strategy, which will help the individuals to protect themselves against influenza virus [89]. An advanced strategy of augmenting the innate and adaptive immune response to infectious disease is to expand the γδ-T cells using phosphoantigens [90,91,92]. In huPBMC sreconstituted immunodeficient Rag2^−/−^γc^−/−^ mice (Table 3) infected with H1N1 influenza A and H5N1 influneza A, phosphoantigen selectively activated and expanded Vδ2-T cells and controlled H1N1 infection, suggesting a new therapeutic approach [93]. 

Among the available vaccines, those that induce memory antibody responses with virus-neutralizing activity are considered the best vaccines against influenza virus infection. Due to the sequence variations in the envelope antigens HA and neuraminidase (NA), multiple subtypes of influenza A virus have evolved and complicated the effective vaccine development. Antigenic drift in the envelope proteins enables H1N1 influneza A and H3N1 influneza A viruses to escape from HA-binding antibodies. As a result, for adults who usually have exposure to seasonal influenza viruses, an annual single dose of the vaccine, boosting the humoral immune response, is sufficient. For emerging influenza viruses, a single dose of a vaccine is sufficient to induce protective immune responses due to the presence of cross-reactive pre-existing CD4^+^ T cells [94]. However, vaccines against H7N9 are less immunogenic [95]. Identification and modification of critical residues in the T cell epitope using immunoinformatics, followed by testing in a humanized (NOD/SCID/Jak3^−^/^−^ (NOJ) mouse, revealed improvements in the HA-binding IgG response, suggesting the utility of a humanized mice model to recapitulate the cross-reactive memory response and subsequent response to the vaccine [96]. Humanized mice can also be used to generate monoclonal antibodies. Humanized mice HLA-A2. HLA-DR4. Rag1KO. IL2rγc KO. NOD, (DRAGA), which lack a murine immune system and express a functional human immune system, were used to develop cross-reactive human anti-influenza monoclonal antibodies (hu-mAb) to study influenza infection and investigate the efficacy of anti-influenza antibody-based therapeutics for human use. The hu mAB that targeted the HA protein of H1N1 was able to clear influenza infection in DRAGA mice [97].

The efficacy of vaccines developed using different strategies, including antiviral DNA vaccines, can be tested in humanized mice models. Ivanova et al. [98] generated anti-influenza IgG antibodies and influenza-specific cytotoxic T lymphocyte (CTL) activity by constructing a chimeric scFv-IP DNA molecule and administering it directly in experimental humanized NOD-SCID gamma mice. The pTriEx-sc22-IPJun/sc22-IP3-Fos chimeric DNA molecule efficiently binds human monocytes and evokes a strong humoral and CTL response in humanized mice [98]. Sasaki et al. [99] succeeded in establishing a humanized mouse model to demonstrate the evaluation of influenza vaccine safety based on the expression of bio marker genes (*ZBP1*, *MX2*, *PSMB9*, *TAP2*, *CXCL11*, *CXCL9*, *TRAFD1*, and *PSME1*) in lungs of the mice model. NOGs engrafted with human PBMCs were used to test NOD/Shi-SCID IL2rγnull mice in the short- and long-term. The results revealed that a short-term reconstitution model of NOD/Shi-SCID IL2rγnul*l* is the most suitable model for biomarker gene-based safety evaluation of vaccines. Human CD14^+^ cells, pDCs, CD4^+^ and CD8^+^ T cells, and B cells were retained in the lungs in the short-term model, while human CD14^+^ cells and pDCs were not detected in the lungs of the long-term model. Moreover, increased levels of human cytokines and chemokines and the expression of human biomarker genes were observed in response to the toxicity of reference vaccines. The observed results suggest that a humanized model can be used for evaluating vaccine safety at the initial level in human peripheral blood mononuclear cells *in vivo* [99].

## 4. Dengue Virus (DENV)

Dengue is a vector-borne viral illness in humans, and it is endemic in more than 100 countries (Available online: http://www.who.int/denguecontrol/epidemiology/en/). Half of the world’s population is at risk for infection. Dengue virus (DENV) belongs to the family *Flaviviridae* and is closely related to hepatitis C, West Nile, Zika, [100] and Japanese encephalitis viruses. These family virus outbreaks are being reported throughout the world (Available online: http://www.who.int/csr/disease/epidemic-focus/flavivirus-epidemics/en/), since they co-circulate and cause similar symptoms, but produce different outcomes. Though several screening and control measures have been developed to detect and differentiate them at an early stage of infection [101], this family of viruses, especially dengue, still poses a great threat to public health. Four different serotypes of dengue have been reported to cause illness in humans (DENV-1-4). They occur concurrently in different parts of the world, and they are the most common viral infection in tropical and sub-tropical countries [102]. Availability of an appropriate small animal model for DENV infection has been, and remains, a challenge. 

### 4.1. Knockout Mouse Models of DENV

At the beginning of the 20th century, researchers struggled to develop an animal model for dengue fever/dengue hemorrhagic fever (DF/DHF) [103]. None of them (small animal models, murine models, and hamsters) have shown any signs of dengue disease. Non-human primate models poorly developed any clinical disease and hence were not a suitable model for DF/DHF. This situation led to the development of different varieties of mouse models. Through cross breeding of mice lacking type I IFN receptors (A129) and those lacking IFN-γ (G129), mice that lacked both the type I and II IFN receptors, AG129 mice (Table 1), were developed in the 1990’s and these mice have become a mainstay of dengue research [104]. Johnson et al. [105] first tested the utility of AG129 mice in DENV vaccine trials. Their study reported that AG129 mice infected with mouse-adapted DENV-2 succumbed to death regardless of age, while immunized mice survived the virus challenge, and survival times increased following passive transfer of the anti-DENV polyclonal antibody. These successful results suggested the utility of AG129 in testing vaccines, as well as antivirals [105]. AG129 mice are now being used extensively in dengue vaccine and antiviral studies [106,107]. C57BL/6 mice lacking only type I IFN receptors succumb to death, even when infected with low doses of a lethal strain of DENV-2 (D220), which was generated by alternatively passaging the virus between C6/36 cells and AG129 mice. C57BL/6 mice lacking only type I IFN receptors in combination with DENV-2 (D220) are being considered as an improved model to study DENV infection and disease and for testing vaccines and antivirals, even under antibody-enhanced infection conditions [108]. Studies on mice with the individual deletion of IFNs (type I & II receptors-IFN-α/β and -γ) and STAT genes have highlighted the role of the STAT-1 and STAT-2 protein in DENV biology [109,110]. Mice lacking either STAT-1 or STAT-2 infected with DENV possess a higher level of viral RNA compared to wild-type mice, but they survive. However, in mice knocked out for both STAT-1 and STAT-2, early death was observed. This demonstrated the role of the STAT2-dependent pathway in mediating transcription of the interferon-stimulated genes against DENV in the absence of STAT-1 [111]. Mice lacking IFN response factors (IRF)-3 and -7 have been shown to be infected with DENV by the bite of infectious mosquitoes; infected mice were also able to transmit the virus to mosquitoes. Hence, this model is being used to understand the transmission dynamics of DENV [112]. Triple KO mice lacking IRF-3, -5, and -7, as well as quadruplet KO mice lacking IRF-1, -3, -5, and -7, have been used to identify the role of IRF-1 in inducing IFN-γ, as well type I IFN, responses against DENV [113]. Moreover, quadruplet KO mice-based experiments have revealed the role of this alternative pathway involving IRF-1 in resistance to DENV [113]. The pathogenic role of CCR5, a chemokine receptor, in supporting viral infection and replication in murine macrophages has been demonstrated in CCR5 KO mice [114]. Even though the use of KO mice has encountered disadvantages, it has contributed enormously to demonstrating the involvement of several cellular signalling pathways in DENV infection. 

### 4.2. Transgenic Mouse Models of DENV

Transgenic mice over-expressing human tumor necrosis factor (TNF)-α have been utilized to demonstrate the role of TNF-α in the development of DENV encephalitis-like symptoms and neurotoxicity. Moreover, anti TNF-α antibodies reduced dengue encephalitis and mortality, suggesting anti TNF-α-based therapeutics for dengue encephalitis [115]. Since mice lacking the IFN-α/β receptor are susceptible to DENV infection and demonstrate T cell responses against DENV infection, studying T cell responses in the context of human MHC molecules might help identify T cell epitopes that are recognized by human T cells [110,116,117,118]. IFNα/βR^−^/^−^ Tg mice (Table 2) expressing human MHC alleles such as HLA, A*0201, A*0101, A*1101, B*0701, and DRB1*0101 have been used to determine the T cell response against DENV. About 42 T cell epitopes have been identified, and most are also recognized by PBMCs from humans. Therefore, Tg mice expressing human MHC alleles might help in identifying T cell epitopes relevant to vaccine design [110].

### 4.3. Humanized Mouse Models of DENV

Human cell responses to DENV infection can be studied better in humanized mouse models since these models provide the opportunity to measure the contribution of host factors in an *in vivo* system. NOD/SCID mice transplanted with human cord blood hematopoietic progenitor (CD34^+^) cells and infected with DENV mimicking natural infection conditions have been used to demonstrate the clinical symptoms of dengue with a fever, rash, and thrombocytopenia [118]. Antibody responses mimicking primary dengue infection have been demonstrated in RAG2^−/−^γ_c_^−/−^ mice that were xenografted with human CD34+ hematopoietic stem cells [119]. A humanized NOD-SCID IL2rγ null mice (Table 3) model has been used to demonstrate differences in the virulence of different DENV-2 genotype strains. Southeast Asian strains produced the highest viremia, followed by Indian strains and Sylvatic strains [120]. An improved humoral response, HLA-A*02-restricted T cell response, and associated IFN-γ production were observed in BLT-NSG mice, but not in NSG mice. Therefore, BLT-NSB mice are an attractive platform for assessing human immune responses to the DENV vaccine [121,122]. However, humanized mice models predominantly develop IgM in response to DENV infection; only a few developed an IgG response because of inefficient class switching, and hence the kinetics of the antibody response was slow [119,123]. Research efforts are expected to lead to the development of ideal models that will show both IgM and IgG responses for studying dengue pathogenesis [124]. The serum metabolomic profile of DENV-2 infected humanized mice has also been utilized to identify the prognostic markers to predict severe dengue. The results yielded a profile similar to those reported in human longitudinal studies [125]. Human immune system (HIS) BLT-NOD/SCID mice have also been demonstrated to be useful in preclinical testing of the efficacy of antiviral drugs against dengue [126]. The following future aspects can be aimed at with the observed results: (i) to test the safety of the DENV vaccine in the recipient without pathology [120,127]; and (ii) to test the immunogenicity of the DENV vaccine [126,128,129].

## 5. Conclusions

Indeed, it is not easy to establish a small animal model for research purposes. An ideal animal model for human viral disease should closely recapitulate the spectrum of clinical symptoms and pathogenesis seen during the course of human infection. Moreover, a perfect animal model should meet other criteria, viz., maximum permissibility to the pathogens, nil resistance to the pathogens, subservience to the same human pathogenic strain, and having the route of infection similar to the human condition. Fulfilling the “Animal rule” of the US Food and Drug Administration [130] is essential for approval and optimally characterized animal models are critical. This rule suits conditions where vaccines and therapeutics cannot be tested safely or ethically on humans; in these cases, approval is possible only after preclinical tests are conducted on animal models. Research in many fields in virology is focussing on standardized models that meet the institutional requirements to evaluate the efficacy of vaccines and therapeutics. In this manuscript, we have summarized available mouse models, such as knockout mice, transgenic mice, and humanized mice models, for HIV-1, influenza, and dengue viruses, and discussed their applications in research. The characters of an ideal animal model cannot always be fulfilled. Since viruses frequently change, mice models need to be changed accordingly, otherwise the natural course of infection will not be accurately mirrored in the animal model. Humanized mice, (CD34+-HSC transplanted immunodeficient mice and BLT mice, NSG, NRG, NOD-SCID) are great models, allowing researchers to recapitulate the key aspects of viral pathogenesis in humans by recreating functional human immune systems in mice. Humanized mouse models are serving as important research tools to experimentally characterize human viral diseases and immune responses. Though humanized mice have advantages over other mouse models, the limitations include: (i) Mouse-to-mouse species variation during the development of a humanized mouse is frequently observed, leading to difficulties in interpreting the results at the end of the experiment; (ii) development of the molecules and cells is not complete in mouse–human chimeric models, and this incompleteness may interrupt the interactions between the cells; (iii) the mechanism of viral pathogenesis is also differently regulated in humanized mice, for example, with regard to B-cell maturation, antibody production, and the coagulation cascade, which are not regulated in the same way [44,130,131,132,133]; (iv) drawbacks include the substantial cost and limited supply and handling of animals in comparison to other models. Despite these limitations, recent developments involving transgenic humanized mice expressing different human genes regulating cellular development are also being attempted [16,18]. Moreover, in traditional KO and Tg mice models, the resulting phenotype due to the knocked out gene or transgenes is expressed during embryonic development itself, and disease onset in these mice models might occur earlier than in humans. To tackle this issue, researchers have developed mice models that can be made to express transgenes when needed using the Cre/lox site specific recombination system. This method provides the opportunity to control gene expression in time and space. These mouse strains express the Cre recombinase enzyme under the control of a promoter that can be induced by drugs such as tetracycline or doxycycline. Then, Cre can activate the expression of transgenes that are flanked by Lox P sites [133,134,135]. Such conditional KO and Tg mice are available for studying cancer and need to be developed for studying viral diseases. With recent advances in genomics, complete information about the mouse genome will serve as an important resource for developing new mice models that will help to completely understand the disease pathogenesis mechanisms and might lead to the discovery of novel therapeutic interventions and prophylactic approaches.

## Figures and Tables

**Table 1:** Knockout mouse models for viral infections.

S.No	Virus	Mouse Model	Name of the Knocked out Gene	Research Application	Reference
1.	HIV	Samhd1 KO (samhd1^−/−^)	Samhd1 deletion.	HIV-1 and HIV-2 entry and pathogenesis	[23,24]
2.	Influenza	C57BL/6J (IL-1R1^−/−^)	Interleukin receptors α &β.	Pathogenesis of influenza.	[75]
		C57BL/6J (IL-81^−/−^)	Interleukin 18	Pathogenesis of influenza.	[76]
		C57BL/6J (cryp^−^/^−^ and casp1^−^/^−^)	Cryopyrin and caspase deficient.	Innate immunity and moderating lung pathology in influenza pneumonia	[77]
		C57BL/6J (FABP5^−/−^)	Deletion of FABP5.	Anti inflammatory response against H1N1 influenza A	[79]
3.	Dengue	129Sv(ev) (AG129 KO)	Type I& II- IFN receptors deficient.	Vaccines research and anti-viral drug design.	[105,106,107]
		STAT1^−/−^ 129/Sv/Ev	Deletion in the DNA binding domain of the STAT1 gene.	STAT1-independent host defense mechanism against viruses	[109,110,111]
		C57BL/6J (Irf3^−/−^x Irf5^−/−^x Irf7^−/−^ triple knockout)	Deletion of interfereon regulators factors (IRF) 3, 5 and 7	Antiviral role of IRF-1 by indcuing IFN responses against DENV infection	[113]

**Table 2:** Transgenic mouse models for viral infections.

S.No	Virus	Mouse Model	Transgene	Application	Reference
1.	HIV	C57BL/6. hu CD4/CCR5 Tg	Human CD4 and chemokine receptor genes	To observe pathological phenotypes of HIV	[31]
		C57BL/6 × C3HF_2_. CD4C/rtTA × TRE/HIVNef) or (CD4C/rtTA2S-M2 × TRE/HIVNef) double-Tg mice upon doxycycline (DOX)	HIV Nef gene	Cellular and molecular pathways of Nef in HIV pathogenesis	[37,38]
		C57BL/6 HIVgp120Tg	HIV gp120	To reveal the role of HIV glycoportein gp120 in binidng to the corecptor CXCR4 in absence of CCR5. CCR5 depletion protects Tg mice against deficits in spatial learning and memory	[41]
		HIV-1 Tg26 transgenic mice	Truncated HIV-1 NL4-3 genome with a 3.1-kb deletion in the Gag and Pol regions	HIV-associated nephropathy	[42]
2.	Influenza	B6.SJL Tg Mice	MX1, MX2, FAM3B and TMPRSS2 genes	Zoonotic transmission of influenza A viruses	[83,84]
		BALB/c. Tg Influenza A HA	Sh-RNAcodes for the knockdown of heamagglutinin	Prevention and control of a viral zoonosis of influenza	[84,85]
3.	Dengue	C57BL/6J Tg HLA-A*02:01 and B10. Tg. HLA-DR3	Geness coding for interspecies hybrid MHC class I molecule of the human HLA-A*0201 allele and the cytoplasmic and transmembrane domains of the mouse H-2Dd class I molecule Genes coding for MHC Class II gene comprising HLA- DR α genomic fragment and a DRB1*030113	To study the CD8+ T cell response to H7N3 influneza A vaccine. Identification of CD4+T cell epitopes for vaccine development	[85,86]

**Table 3:** Humanized mouse models for viral infections.

S.No	Virus	Mouse Model	Humanization	Application	Reference
1.	HIV	Hu-PBL-SCID mice	SCID mice populated with human peripheral blood leukocytes	HIV infection, replication and pathogenesis	[16]
		HSCs-BLT mice, NOD-SCID BLT and NSG-BLT.	HSCs-mice engrafted and bone liver/thymus	Human disease pathogenesis, retroviral spread and restored CD4^+^ and CD8^+^ T cell numbers on ART treatment	[43,44,45,46,47,48]
		C57BL/6 *Rag2^−/−^ γc ^−/−^CD47^−/−^* triple knockout (TKO)-BLT mouse.	Xenotransplantation with human immune system	HIV-latency	[50]
		NOD/ SCID (NS), NOD/SCID IL2rgc^−/−^ (NSG) or NOD/Rag1^−/−^ IL2rgc^−/−^ (NRG)	Reconstitution of different types of human tissues	Treatment of systemic HIV infection with ART and HIV latency	[45,46,47,48]
2.	Influenza	C57BL/10SgAiRag2^−/−^γc^−/−^ mice.	Humanized with huPBMCs	Vaccine based studies and therapeutics for human pathogens	[93]
		DRAGA mouse; HLA-A2. HLADR4. Rag1KO. IL-2Rgc KO. NOD.	Humanized with functional human immune system	Anti-influenza monoclonal antibodies	[97]
		NOD/Shi-SCID-IL2rγnull (NOG).	Humanized with huPBMCs.	Evaluating vaccine safety	[99]
3.	Dengue	RAG2^−/−^γ_c_^−/−^ mice	Xenografted with human CD34+ hematopoietic stem cells	Antibody responses against DENV	[119]
		NOD-SCID IL2rγ null	Transplanation of purified cord blood CD34+ cells	To demonstrate differences in the virulence of different DENV-2 strains	[120]
		HIS BLT-NOD/SCID mice	Human immune system	Preclinical testing of antiviral drugs against dengue	[127]
